# Restoration of NOX4 signalling reverses endothelial colony-forming cell angiogenic dysfunction associated with experimental and clinical diabetes

**DOI:** 10.1186/s13287-025-04393-4

**Published:** 2025-06-02

**Authors:** Karla M. O’Neill, Kevin S. Edgar, Shun Hay Pun, David C. Campbell, Tinrui Toh, Xin N. Wong, Bianca Botezatu, Jyoti Kandel, Una McCoy, Jennifer Nicell, Catherine McClintock, Kiran J. McLoughlin, Yuxin Wu, Vinuthna Vani Madishetti, Arya Moez, Mohammed Alsaggaf, Eleanor K. Gill, Rawan A. Abudalo, Christina L. O’Neill, Edoardo Pedrini, Jasenka Guduric-Fuchs, Coy Brunssen, Henning Morawietz, Philip D. Dunne, Chris J. Watson, Reinhold J. Medina, David J. Grieve

**Affiliations:** 1 Wellcome-Wolfson Institute for Experimental Medicine, Queen’s University Belfast, Belfast, UK; 2 Patrick G Johnston Centre for Cancer Research, Queen’s University Belfast, Belfast, UK; 3 Division of Vascular Endothelium and Microcirculation, Dresden University of Technology, Dresden, Germany; 4 Faculty of Pharmaceutical Sciences, The Hashemite University, Zarqa, Jordan

**Keywords:** NADPH oxidase 4, Endothelial progenitor cells, Angiogenesis, Diabetes cardiovascular diseases

## Abstract

**Background:**

Progenitor endothelial colony forming cells (ECFCs) are critical for vascular homeostasis and hold therapeutic potential for ischaemic cardiovascular disease (CVD). As angiogenic capacity and efficacy within diseased tissues is particularly impacted in diabetic patients, who show high incidence of ischaemic CVD, targeting of critical ECFC pathways in this setting represents an innovative focus towards enhancing intrinsic vasoreparative function. We previously reported that NADPH oxidase 4 (NOX4)-derived reactive oxygen species promote cord blood-derived ECFC (CB-ECFC) pro-angiogenic response, whilst NOX4 overexpression (OE) enhances revascularisation capacity. Here, we aimed to investigate specific influence of NOX4-dependent signalling on CB-ECFC angiogenic dysfunction observed upon exposure to both experimental and clinical diabetes to define whether NOX4 may represent a viable therapeutic target in this context.

**Methods:**

CB-ECFCs were cultured in high glucose (D-glucose, 25 mmol/L) or control media (5 mmol/L) ± phorbol 12-myristate 13- acetate (PMA, 500 nmol/L) for 72 h with assessment of migratory/tubulogenic capacity and NOX4 mRNA expression (qRT-PCR). Detailed analysis of angiogenic function and signalling (Western blot, RNA sequencing) was performed in CB-ECFCs isolated from donors with gestational diabetes prior to NOX4 plasmid OE to define rescue potential and key mechanistic pathways (network analysis, proteome profiling). Statistical significance was determined using one-way ANOVA with Bonferroni post-host testing or paired/unpaired Student’s t-test, as appropriate.

**Results:**

PMA-stimulated CB-ECFC migration and tube-forming capacity observed in control cells was suppressed in experimental diabetes in parallel with reduced *NOX4* expression and rescued by plasmid NOX4OE. As direct evidence of clinical relevance, CB-ECFCs from gestational diabetic donors showed reduced angiogenic potential associated with attenuated NOX4, eNOS activity and downregulation of key vasoreparative signalling. Furthermore, NOX4OE rescued angiogenic function in chronically diabetic CB-ECFCs via modulation of downstream signalling involving both direct and indirect enhancement of pro-angiogenic protein expression (endoglin/SERPINE1/E2F1) linked to reduced p53 phosphorylation.

**Conclusions:**

Taken together, these data indicate for the first time that reduced NOX4 expression plays a pivotal role in CB-ECFC angiogenic dysfunction linked with diabetes whilst highlighting NOX4-dependent signalling as a potential target to protect and augment their intrinsic vasoreparative capacity towards addressing current translational barriers.

## Introduction

Angiogenesis is defined as growth of new blood vessels following proliferation, migration and remodelling of fully differentiated endothelial cells (EC) derived from pre-existing parent vessels [1]. Angiogenic dysfunction is a key pathological driver of ischaemic cardiovascular disease (CVD) development and progression, with associated conditions such as heart disease, stroke and peripheral arterial disease remaining a major global health burden [2, 3]. Whilst effective CVD management continues to represent a significant clinical challenge, focus has been directed towards defining therapeutic potential of novel cell-based approaches to promote critical revascularisation processes and hypoxic tissue repair [4–7]. In this regard, endothelial colony forming cells (ECFCs) are a defined progenitor subset, which circulate in the blood and rapidly home to sites of ischaemia, and possess proven angiogenic capability both in vitro and in vivo, underlining their established role in vascular homeostasis and repair [8–13]. Whilst ECFCs are phenotypically equivalent to mature ECs in vitro, they proliferate faster and possess superior angiogenic capacity [10, 14], with our previous studies demonstrating that they fully integrate with the microvasculature in vitro and promote neovascularisation in vivo via regulation of pro-angiogenic paracrine signalling [9, 10]. Importantly, ECFC administration in experimental models of human ischaemic disease, corresponding to various tissue sources (brain, limb, myocardium, retina), underlines their prominent role in revascularisation and evident therapeutic potential [9–12].

Despite clear promise, major translational barriers to ECFC therapy remain, such as insufficient efficacy in the CVD microenvironment and impaired angiogenic capacity of ECFCs isolated from ischaemic CVD and diabetic patients [15–17]. Characterised as an initially progressive and then chronic metabolic disease, diabetes impacts both mature EC and ECFC angiogenic potential, largely due to hyperglycaemia, resulting in poor vascular homeostasis, attenuated vasoreparative capacity, and ischaemic CVD [15, 16, 18]. Dysfunctional ECs may also promote atherosclerotic plaque development as a critical determinant of diabetic co-morbidities associated with underlying tissue or organ-specific microvascular dysfunction [19]. Given that the global incidence of diabetes has reached epidemic proportions and is predicted to affect > 700 million individuals by 2045, there is urgent need to develop alternate approaches for effective clinical management and treatment of ischaemic CVD [20], which disproportionately impacts diabetic patients who typically experience accelerated progression and poor outcomes [21].

In this regard, cord blood-derived ECFCs (CB-ECFCs) appear to represent the most potent and least immunogenic cell-based source for allogenic therapy. Although much research has focused on enhancing intrinsic angiogenic function of autologous ECFCs isolated from diabetic patients, utilising pharmacological or genetic approaches towards development of personalised cell therapy [16, 22–25], this source is problematic due to extended in vitro expansion and persistent dysfunction. Therefore, recent attention has focused on CB-ECFCs as a more readily available source with low immunogenicity, whilst also harnessing vasoreparative function of endogenous circulating ECFCs which is reduced in diabetes [26]. Detailed understanding of key mechanisms and signalling pathways underpinning diabetes associated ECFC angiogenic dysfunction is required to support advancement of ECFC-based therapeutic approaches. Such knowledge will inform identification of critical signalling pathways which may be targeted to either rescue pro-angiogenic capacity of endogenous diabetic ECFCs or protect function of exogenous healthy or repaired CB-ECFCs upon introduction to the diabetic tissue microenvironment [25–27].

It is well established that NADPH-oxidase derived-reactive oxygen species (ROS) are an important regulator of angiogenic signalling [28], whilst we have identified NADPH oxidase 4 (NOX4) as a major determinant of physiological CB-ECFC function, supporting creation of a pro-angiogenic environment and enhanced ability of CB-ECFCs to promote in vitro vascular network formation and in vivo revascularisation [10]. Consequently, we hypothesised that dysregulated NOX4 signalling may underlie reduced angiogenic capacity of diabetic CB-ECFCs via modulation of important cellular processes. The aim of this study was to specifically investigate impact of experimental and clinical diabetes on CB-ECFC angiogenic function in relation to NOX4 signalling towards identification of key mediators as candidates for selective targeting to enhance therapeutic efficacy in this setting. Here, we report for the first time, an important role for NOX4 signalling in regulating CB-ECFC pro-angiogenic response in high-glucose conditions. Specifically, we show that ability of CB-ECFCs to respond to phorbol 12-myristate-13-acetate (PMA) as an angiogenic stimulus [29] is suppressed by exposure to experimental diabetes, whilst angiogenic potential and vasoreparative signalling is reduced in CB-ECFCs isolated from donors with gestational diabetes as direct evidence of clinical relevance. Restoration of NOX4 protein levels via plasmid overexpression (OE) reversed angiogenic dysfunction in chronically diabetic CB-ECFCs via upregulation of key pro-angiogenic signalling involving endoglin, SERPINE1 and the pro-proliferative transcription factor, E2F1 [30]. NOX4OE CB-ECFCs also showed reduced p53 phosphorylation (S46), with network analysis revealing linkage to downstream positive regulation of endoglin and E2F1 expression, indicating that NOX4-mediated inhibition of p53 activation may heighten ECFC resilience to hyperglycaemia-mediated and p53-dependent cell cycle arrest, apoptosis and senescence [31–33]. Taken together, these data clearly support a significant role for NOX4 in regulating CB-ECFC dysfunction in diabetes, whilst highlighting selective targeting of downstream angiogenic signalling as a potential innovative approach to augment therapeutic efficacy of both endogenous circulating ECFCs and allogeneic CB-ECFCs in this setting.

## Materials and methods

### Cell culture

Individual CB-ECFC colonies were isolated from fresh umbilical cord blood (obtained from healthy or gestational diabetic donors) under local ethical approval (National Research Ethics Service Committee 15/YH/0281) prior to immunophenotyping by flow cytometry and maintenance in Endothelial Growth Basal Medium-2 (EGM2, supplemented with BulletKit without antibiotics; Lonza) and 12% Foetal Bovine Serum (FBS; Sigma). All culture plates were coated with type 1 collagen (Corning) for 1 h before CB-ECFC incubation at 37 °C with 5% CO_2_. For all experiments, CB-ECFCs from multiple donors were utilised between passage 6–12.

### Genetic manipulation and cell treatments

For NOX4OE, up to 5 × 10^5^ CB-ECFCs were electroporated using the Amaxa® system and associated Basic Nucleofector Kit for Primary Mammalian Endothelial Cells, according to manufacturer’s instructions (Lonza). A total of 1 µg of pcDNA4/TO/myc-His A (empty vector, EV) or pcDNA4/TO/NOX4-myc-His expression vector containing a full length myc-tagged copy of NOX4 was introduced into cells [10]. For high glucose studies, CB-ECFCs were cultured in 12% EGM2 containing 25 mmol/L D-glucose for 72 h prior to treatment with PMA (500 nmol/L; Sigma) for 24 h in Dulbecco's Modified Eagle's medium (DMEM; Sigma) to prevent confounding effects of antioxidants in EGM2.

### Cell viability

For cell viability assessment, 1 × 10^4^ CB-ECFCs were seeded on 96-well plates and left to attach overnight in 12% FBS-containing EGM2 media. Media was subsequently replaced with fresh 12% EGM2 containing 0.5 mg/ml MTT (Sigma) prior to incubation for 3 h at 37 °C. Following washing and DMSO treatment (30 min, 37 °C), absorbance was measured (570 nm) to determine formazan crystal production by viable cells.

### Cell migration

CB-ECFCs were cultured on 6-well plates until they reached 80–90% confluency. A scratch was introduced on the monolayer using a sterile tip before CB-ECFCs were incubated for a further 24 h in the presence of appropriate media/treatment. A light microscope (Leica DM2000 LED) was used to take 3 images (to provide an average equating to n = 1) per scratch (20X) and ImageJ software (NIH) used to calculate migration into the cell free zone.

### Cell proliferation

CB-ECFCs (1 × 10^5^) were seeded on T75 flasks and left to attach overnight. Cells were cultured for a further 48 h before being trypsinised and counted using a haemocytometer. Ability of cells to proliferate during this time was determined by calculating number of cells/ml.

### Tubulogenesis

For 2D tubulogenesis assay, 48-well plates were coated with growth-factor reduced Matrigel (150 µl/well; Corning) and incubated for 30 min at 37 °C to allow polymerisation prior to seeding of 5 × 10^4^ CB-ECFCs resuspended in the appropriate EBM2 ± treatment. Following 24 h incubation at 37 °C, tube networks were imaged with a light microscope for quantification of tube length and branch number using ImageJ based on the average of 3 images (Leica DM2000 LED, 20X magnification) equating to n = 1. For 3D tubulogenesis assay, 6 × 10^4^ CB-ECFCs were resuspended in growth-factor reduced Matrigel (Corning) mixed 60:40 with complete 12% EGM2 and 40 µl blobs spotted onto 24-well plates, before being left at room temperature for 10 min. Plates were incubated for a further 20 min at 37 °C to polymerise before addition of 1 ml complete media and incubation for 48 h. Blobs were then stained with calcein (2 µg/ml in Hank’s Balanced Salt Solution, HBSS; Thermo Fisher Scientific) for 1 h at 37 °C before transfer to fresh HBSS and imaging using a Nikon Eclipse TE2000-U inverted confocal microscope. A total of 5 images were taken per blob to ensure adequate coverage of the 3D network and ImageJ used for quantification of area of fluorescence (taken to denote n = 1; 15 images).

### RNA analysis

Total RNA was extracted using High Pure RNA isolation kit (Roche) according to manufacturer’s instructions. After confirmation of concentration and purity by NanoDrop, 500–1000 ng RNA was converted to cDNA using High-Capacity cDNA Reverse Transcription Kit (Thermo Fisher Scientific) and various target genes amplified using quantitative RT-PCR (qRT-PCR; SYBR Green detection, Roche). Gene-specific primers (IDT) were designed using UCSC genome browser (spanning an intron where possible) and validated for use by qRT-PCR via the standard curve method. Relative quantification values were obtained using the ∆∆Ct method. Primer sequences (5′-3′): *NOX4* FW (ATGGTGGTGGTGCTATTCCT), *NOX4* RV (CTGAAACATGCAACGTCAGC); *HSP90AB1* FW (GCGCTCTCATACCTCCCAGT), *HSP90AB1* RV (CAAAAGCTGAGTTGGCCCAC).

### Flow cytometry

For immunophenotyping, respective isotype controls were used to determine baseline voltages for PMT detectors for each fluorophore before data acquisition. Immunophenotyping was carried out using the following antibodies: anti-human CD31-PE (12-0319-42, eBioscience), anti-human CD105-PE (12-1057-42, eBioscience), anti-human CD45-eFluor450 (48-0459-42, eBioscience), anti-human CD90-FITC (11-0909-42, eBioscience), mouse IgG1-PE (12-4714-42, eBioscience), mouse IgG1-eFluor450 (48-4714-82, eBioscience), mouse IgG1-FITC (11-4714-42, eBioscience). Analysis of flow cytometry data was carried out using FlowJo v10.9 (Beckton-Dickinson, USA).

### Western blotting

CB-ECFCs were lysed using protein extraction RIPA buffer (50 mmol/L Tris–HCl, pH 8.0; 150 mmol/L sodium chloride; 1.0% Igepal; 0.5% sodium deoxycholate; 0.1% sodium dodecyl sulphate; Sigma) containing protease inhibitor cocktail (Roche) and phosphatase inhibitor (Thermo Fisher Scientific) prior to quantification using BCA assay (Thermo Fisher Scientific). Up to 40 µg protein was separated on a 10% SDS–polyacrylamide gel prior to electro-transfer onto PVDF membrane (GE Healthcare) before blocking for 1 h at room temperature with 5% non-fat milk in Tris-buffered saline with 0.1% Tween-20 (TBS-T). Membranes were then incubated overnight at 4 °C with primary antibodies against ACTB (#3700, Cell Signaling Technology, 1:5000), NOX2 (ab80508, Abcam, 1:2000), NOX4 (ab133303, Abcam, 1:2000), VEGF Receptor 2 (55B11, Cell Signaling Technology, 1:1000), Phospho-VEGF Receptor 2 (Try 1175; D5B11, Cell Signaling Technology, 1:1000), eNOS (610297, BD Transduction Labs, 1:500), Phospho-eNOS (S1177, 9571S Cell Signaling Technology, 1:1000), SERPINE1 (66261-1-Ig, Proteintech, 1:10000), Endoglin (#14606, Cell Signaling Technology, 1:1000) and E2F1 (66515-1-Ig, Proteintech, 1:3000). Following washing (× 3, TBS-T), membranes were incubated with their respective HRP-conjugated secondary antibodies (#7074, #7076, Cell Signaling Technology, 1:5000) for 1 h at room temperature before further washing (×3, TBS-T), and detection of protein bands using chemiluminescent HRP Substrate (Merck) and quantification using ImageJ.

### Human proteome profiling

Proteome profiler arrays (R&D Systems) specific for protein phosphorylation (Human phospho-kinase proteome profiler array, ARY003C) or linked with angiogenesis (Human angiogenesis proteome profiler array; ARY007) were run according to manufacturer’s instructions. Equal concentrations of CB-ECFC lysate were pooled (300 µg, 6 clones in triplicate, EV and OE) and incubated with membranes pre-loaded with antibodies for multiple targets. Pixel density of each spot was determined using HLImage++ software (Western Vision Software) and duplicate spots averaged and normalised to reference spots. Differential expression was presented as heat maps, generated using Microsoft Excel (increases shown in green, decreases shown in red) relative to EV control.

### Pathway and network interrogation

RNA sequencing was carried out on both healthy and diabetic CB-ECFC clones using the NextSeq 2000 platform. Further to alignment and count generation, statistical analysis (using analysis of variance/ANOVA), differential gene expression generation (DESeq2) and visualisation of the data (volcano plot, heatmap, Gene Set Enrichment Analysis/GSEA) were performed using Bioconductor package (pheatmap and MouS^R^ (https://mousr.qub.ac.uk/). The resultant gene list was uploaded to Ingenuity Pathway Analysis (IPA, Qiagen) for identification of key dysregulated pathways and NOX4 dependent networks based on log2 fold change ± 0.58 and *P* < 0.05. KEGG analysis via Enrichr (https://maayanlab.cloud/Enrichr/) was used to further interrogate impact of ECFC exposure to gestational diabetes on NOX4 signalling (Biological process/BP).

### Statistical analysis

Data were analysed using GraphPad Prism software and expressed as mean ± SEM. When comparing between multiple groups, statistical significance was calculated using one-way ANOVA with Bonferroni post-host testing. Either paired or unpaired Student’s t-test was performed when comparing two groups. *P* < *0.05* was taken to indicate statistical significance after Grubb’s test analysis (https://www.graphpad.com/quickcalcs/grubbs1/) for exclusion of outliers.

## Results

### PMA-induced CB-ECFC pro-angiogenic response and NOX4 activation is suppressed in experimental diabetes

**Fig. 1:**
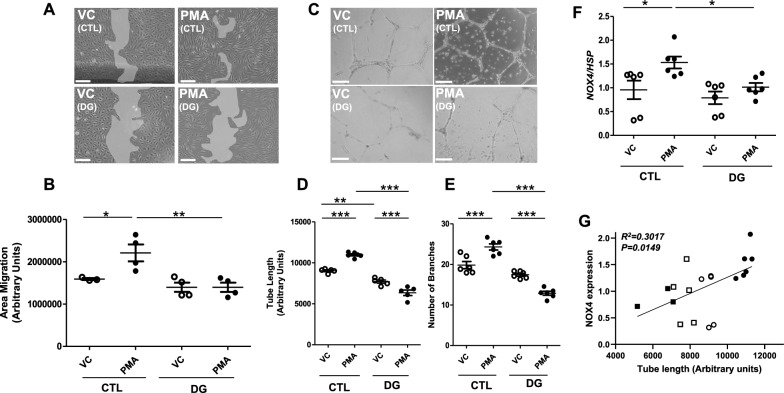
**PMA-induced pro-angiogenic CB-ECFC function and NOX4 activation is suppressed in experimental diabetes. **CB-ECFCs from healthy donors were cultured in normal (CTL/control, 5 mmol/L) or high glucose (DG, 25 mmol/L) for 72 h followed by 24 h treatment with either vehicle control (VC) or phorbol 12-myristate-13-acetate (PMA, 500 nmol/L). **A**, **B** Scratch-wound assay over 16 h to determine cell migration; n = 3–4, combined data from three different CB-ECFC clones with representative images shown from a single clone for each treatment group. **C**–**E** 2D Matrigel tubulogenesis assay over 24 h with quantification of tube length and branch number; n = 5–6, combined data from three different CB-ECFC clones with representative images shown from a single clone for each treatment group. **F** qRT-PCR analysis of *NOX4* mRNA expression relative to *HSP90AB1*; n = 6, combined data from three different clones. **G** Correlation analysis of tube length (**D**) versus NOX4 expression (**F**) with both P and R^2^ values indicated. Data are mean ± SEM; *P < 0.05, **P < 0.01, ***P < 0.001, one-way ANOVA with Bonferroni post-hoc testing

To determine impact of experimental diabetes on pro-angiogenic response, CB-ECFCs were isolated from healthy donors and cultured under normal glucose (5 mmol/L) or high glucose conditions (25 mmol/L; 72 h) prior to exposure to vehicle control (VC) or PMA (500 nmol/L), which we have shown to promote CB-ECFC angiogenic capacity via PKC-mediated superoxide generation [10, 34]. As previously reported, PMA augmented CB-ECFC migration and tube formation (scratch wound and 2D tubulogenesis assay, respectively) under control conditions (Fig. 1A–E). Although 72 h exposure to high glucose did not impact basal CB-ECFC angiogenic function, PMA-induced migration was abolished (Fig. 1A, B) whilst tube formation (tube length and branch number) was further reduced when comparing PMA treatment versus VC (Fig. 1C, E). Consistent with our previous report that CB-ECFC pro-angiogenic response is mediated by and enhanced by NOX4-dependent signalling, NOX4 mRNA expression (qRT-PCR) was augmented by PMA treatment under normal glucose conditions, but not after exposure to elevated glucose (Fig. 1F), and was positively correlated with tube length (Fig. 1G). Taken together, these data clearly indicate that experimental diabetes limits CB-ECFC pro-angiogenic capacity, whilst suggesting that loss of NOX4 signalling may be pivotal in mediating the observed lack of migratory and tubulogenic response.

### NOX4 overexpression restores CB-ECFC pro-angiogenic response to PMA stimulation in experimental diabetes

**Fig. 2:**
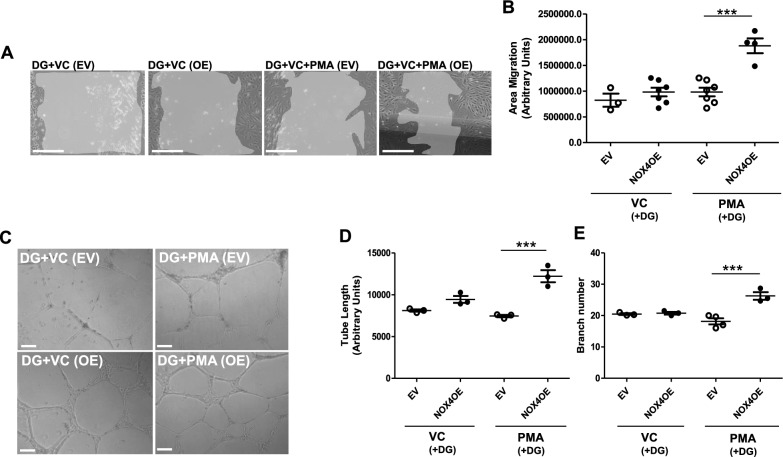
**NOX4 overexpression restores CB-ECFC pro-angiogenic response to PMA stimulation in experimental diabetes.** CB-ECFCs from healthy donors were subjected to electroporation for introduction of either empty vector (EV; pcDNA4/TO/myc-His A) or NOX4 overexpression (OE; pcDNA4/TO/NOX4-myc-His A) construct prior to culture in high glucose (DG, 25 mmol/L) for 72 h and treatment with vehicle control (VC) or phorbol 12-myristate-13-acetate (PMA). **A**, **B** Scratch-wound assay over 16 h to determine cell migration in response to vehicle control (VC) or phorbol 12-myristate-13-acetate (PMA); n = 3–7, combined data from three different clones with representative images shown from a single clone for each treatment group. **C**–**E** 2D Matrigel tubulogenesis assay over 24 h with quantification of tube length and branch number; n = 3–4, combined data from three different clones, with representative images shown from a single clone. Data are mean ± SEM; ***P < 0.001, one-way ANOVA with Bonferroni post-hoc testing

Having highlighted NOX4 signalling as a likely important regulator of angiogenic response in healthy CB-ECFCs exposed to experimental diabetes, we investigated whether migratory and tubulogenic function could be rescued by NOX4OE in this setting. CB-ECFCs were electroporated for introduction of OE plasmid containing a full-length copy of NOX4 cDNA or control EV [10]. Whilst migration and tube formation (assessed as tube length and branch number) of CB-ECFCs exposed to high glucose and EV (which showed preserved angiogenic function; Fig. 1A–E) were not impacted by PMA stimulation, cells subjected to NOX4OE showed markedly enhanced migration (Fig. 2A, B) and tube formation (Fig. 2C–E) with PMA treatment. These data indicate that induced NOX4 expression restores suppressed CB-ECFC pro-angiogenic response in experimental diabetes, highlighting this major ROS source as a potential target to protect or enhance CB-ECFC function in this context.

### CB-ECFCs exposed to clinical diabetes display impaired pro-angiogenic capacity and signalling in parallel with reduced NOX4 expression

**Fig. 3:**
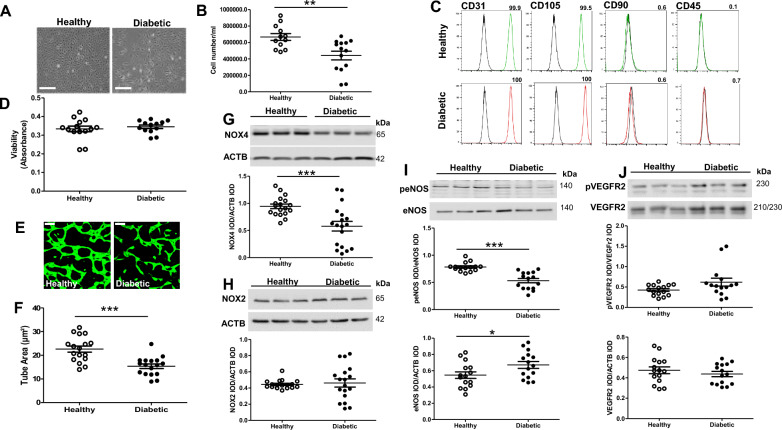
**CB-ECFCs exposed to clinical diabetes display impaired angiogenesis and signalling linked with reduced NOX4 expression.** Characterisation of CB-ECFCs isolated from healthy and gestational diabetic donors. **A**, **B** Proliferation assay by counting number of cells/ml at 48 h post-seeding; n = 12–14, combined data from 5–6 different clones; representative light microscope images at 10X magnification. **C** Immunophenotyping by flow cytometry to confirm absence of mesenchymal/haematopoietic markers (CD90, CD45) and presence of EC markers (CD105, CD31); n = 4, data collected from 4 different clones, representative histograms shown with % positive cells for each marker displayed above each panel. **D** MTT cell viability assay; n = 13–14, combined data from 6 different clones. **E**, **F** 3D Matrigel tubulogenesis assay over 48 h with quantification of tube area; n = 17, combined data from 6 different clones with representative images shown from one healthy and diabetic clone (10X magnification). Protein expression of **G** NOX4, **H** NOX2, **I** phospho-eNOS (peNOS) and total eNOS, and **J** phospho-VEGFR2 (peVEGFR2) and total VEGFR2 by Western blotting with normalisation to ACTB as loading control; n = 15–18, combined data from 6 different clones (NOX4 and NOX2); representative cropped blots shown from one healthy and one diabetic clone. Full-length blots/gels are presented in Supplementary Figure. Data are mean ± SEM; *P < 0.05, **P < 0.01, ***P < 0.001, unpaired Student’s *t*-test

It is well established that chronic exposure of mature and progenitor ECs, including ECFCs, to high glucose promotes angiogenic dysfunction [16, 35]. Having identified a likely important role for NOX4 in mediating CB-ECFC stimulated pro-angiogenic response in experimental diabetes, we isolated CB-ECFCs from donors with gestational diabetes as a more clinically relevant model of chronic disease to assess NOX4 impact. Characterisation of diabetic CB-ECFCs indicated that whilst proliferation was reduced (Fig. 3A, B), they exhibited equivalent immunophenotype compared to healthy CB-ECFCs (Fig. 3C), characterised by positive expression of CD31 and CD105 (EC markers) and negative expression of CD90 (mesenchymal marker) and CD45 (haematopoietic marker), and similar viability measured by MTT (Fig. 3D). In contrast to 72 h high glucose exposure, tube formation capacity (3D Matrigel assay) of CB-ECFCs exposed to longer-term clinical diabetes was impaired (Fig. 3E, F), reflected by reduced tube area and inability to form complete and extensive networks. Similarly, whilst CB-ECFCs exposed to experimental diabetes failed to induce NOX4 upon stimulation, cells from gestational diabetic donors showed reduced NOX4 protein expression compared with those from healthy donors (Fig. 3G), whilst NOX2 levels were not impacted (Fig. 3H). Reduced angiogenic capacity observed in chronically diabetic CB-ECFCs was associated with decreased eNOS activity, reflected by reduced phosphorylation at S1117, despite elevated total eNOS protein (Fig. 3I), although VEGFR2 expression and phosphorylation remained unaltered in diabetic versus healthy CB-ECFCs (Fig. 3J). Taken together, these data further support NOX4-dependent signalling as a key determinant of CB-ECFC angiogenic function in diabetes.

### Clinical diabetes induces dysregulation of CB-ECFC transcription linked with key angiogenic signalling pathways

**Fig. 4:**
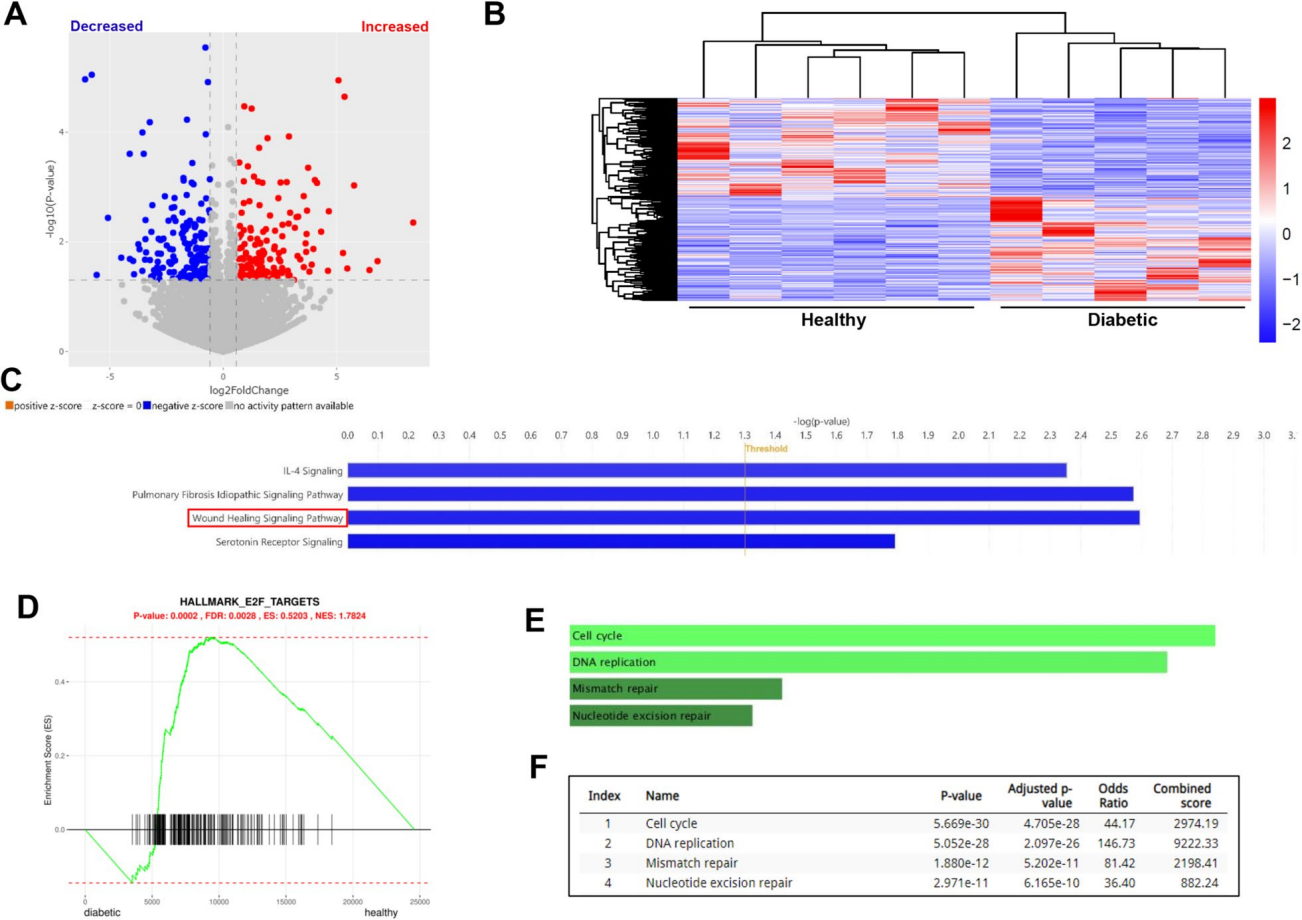
**CB-ECFCs exposed to clinical diabetes display altered transcription profile with dysregulation of key angiogenesis-linked pathways. **CB-ECFCs were isolated from healthy and gestational diabetic donors and subjected to RNA sequencing prior to bioinformatics analysis with visualisation of differential expression by **A** volcano plot (MouS^R^) and **B** heatmap (RStudio; 641 genes, *P* < 0.05). **C** Ingenuity pathway analysis (IPA) of healthy and diabetic CB-ECFCs based on defined thresholds (log2 fold change ± 0.58, *p* < 0.05) to identify top down-regulated pathways (z-score > − 2); shown in blue (threshold –log *p* > 1.3). **D** Gene Set Enrichment Analysis (GSEA) identified ‘E2F targets’ as being significantly enriched in diabetic versus healthy CB-ECFCs (ES, enrichment score: 0.5203; FDR, false discovery rate: 0.0028). KEGG analysis (KEGG 2021 Human) by Enrichr using extracted GSEA (‘E2F targets’) list to group genes according to biological process with identified pathways presented as **E** bar graph, and **F** table (top 4 including *P* value and FDR)

RNA sequencing of CB-ECFCs isolated from healthy donors and those with gestational diabetes was conducted to determine key genes and pathways underlying observed angiogenic dysfunction. Further to alignment and generation of a differentially expressed gene list, significantly up- and down-regulated genes (which did not include NOX4) were visualised via generation of both a volcano plot (Fig. 4A; MouS^R^) and heat-map (Fig. 4B; RStudio). Downregulated genes (considered to be most functionally relevant) were then uploaded to Ingenuity Pathway Analysis (IPA) to enable identification and ranking of linked pathways which were altered within the dataset. Amongst differentially regulated signalling pathways, wound healing was highlighted as one of the most significantly downregulated in diabetic versus healthy CB-ECFCs (Fig. 4C). Interrogation of impacted signalling pathways in diabetic CB-ECFCs, using Gene Set Enrichment Analysis (GSEA) to determine over-represented gene sets, highlighted significant enrichment of genes targeted by the E2F family of transcription factors in diabetic CB-ECFCs (Fig. 4D). Consistent with known functions of E2F signalling, KEGG assessment of the extracted gene list using Enrichr linked associated genes with positive regulation of cell cycle progression and DNA replication, in addition to mismatch repair and nucleotide excision repair (Fig. 4E, F) [36].

### NOX4 overexpression promotes functional rescue of CB-ECFCs exposed to clinical diabetes via induction of pro-angiogenic signalling

**Fig. 5:**
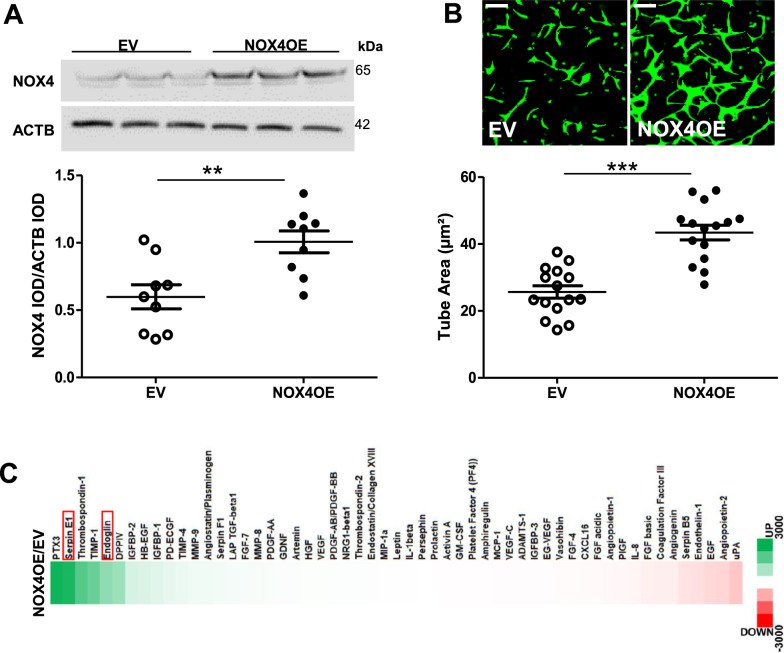
**NOX4 overexpression promotes functional rescue of CB-ECFCs exposed to clinical diabetes by inducing pro-angiogenic signalling. **CB-ECFCs from gestational diabetic donors were subjected to electroporation for introduction of either empty vector (EV; pcDNA4/TO/myc-His A) or NOX4 overexpression (OE; pcDNA4/TO/NOX4-myc-His A) construct. **A** Confirmation of NOX4 protein expression at 48 h post-electroporation; n = 9, combined data over 3 different clones, with representative cropped blot shown from a single clone. **B** 3D Matrigel tubulogenesis assay over 48 h with quantification of tube area; n = 15, combined data from 5 different clones with representative images shown from a single clone (10X magnification). **C** Proteome Profiler™ analysis of pooled lysates (triplicates from 5 clones) for detection of angiogenesis-linked proteins. Differential expression between groups presented as a heat map indicating increased (green) and decreased (red) expression (OE versus EV). Full-length blots/gels are presented in Supplementary Figure. Data are mean ± SEM; **P < 0.01, ***P < 0.001, paired Student’s *t*-test

Given that NOX4 protein expression (but not mRNA) was decreased in diabetic CB-ECFCs in parallel with reduced angiogenic capacity, we hypothesised that restoration of NOX4 signalling would reinstate healthy tubulogenic function. This was addressed by electroporation of NOX4OE or EV plasmid into diabetic CB-ECFCs, with confirmation of increased NOX4 protein expression at 48 h post-transfection (Fig. 5A), prior to seeding on Matrigel for 48 h for 3D tubulogenesis assay. CB-ECFCs isolated from donors with gestational diabetes and subjected to NOX4OE showed improved ability to form tube networks versus those transfected with EV, quantified as increased tube area (Fig. 5B). Complementary proteome profiler analysis of protein lysates highlighted differential expression of key angiogenic mediators (Fig. 5C), visualised as a heat map indicating marked upregulation (green) of proteins associated with pro-angiogenic response in NOX4OE versus EV diabetic CB-ECFCs in parallel with downregulation (red) of known negative regulators of angiogenesis. Of the top differentially altered proteins linked with NOX4OE, endoglin and SERPINE1 (outlined in red) were considered to be most relevant to CB-ECFC function in diabetes due to their established pro-angiogenic actions [37, 38].

### Clinical diabetes induces dysregulation of CB-ECFC angiogenic pathways linked with downstream NOX4-dependent pro-angiogenic signalling

**Fig. 6:**
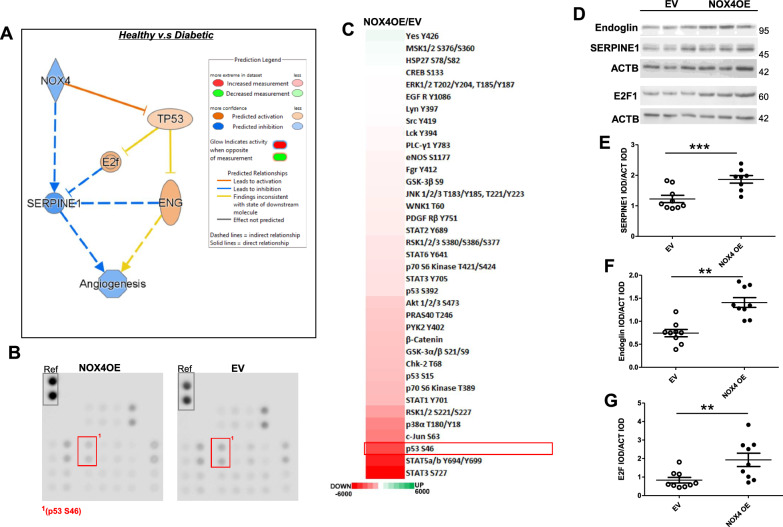
**Clinical diabetes induces dysregulation of CB-ECFC angiogenic pathways linked with downstream NOX4-dependent pro-angiogenic signalling. **CB-ECFCs were isolated from healthy and gestational diabetic donors and subjected to RNA sequencing prior to (**A**) IPA network generation, node colour represents predicted gene activation (orange) and inhibition (blue). **B**, **C** Proteome Profiler™ analysis of pooled lysates (triplicates from 5 clones) from diabetic CB-ECFCs subjected to electroporation for introduction of either empty vector (EV; pcDNA4/TO/myc-His A) or NOX4 overexpression (OE; pcDNA4/TO/NOX4-myc-His A) construct for detection of protein phosphorylation. Differential expression between groups presented as a heat map indicating largely decreased (red) phosphorylation (OE versus EV). **D**–**G** Protein expression of endoglin, E2F1 and SERPINE1 by Western blotting with normalisation to ACTB as loading control; n = 8/9, combined data from 3 different clones. Representative cropped blots shown from one healthy and one diabetic clone. Full-length blots/gels are presented in Supplementary Figure. Data are mean ± SEM; **P < 0.01, ***P < 0.001, paired Student’s *t*-test

Further to our finding that NOX4OE promoted rescue of reduced angiogenic capacity in diabetic CB-ECFCs (Fig. 5B), detailed network analysis was carried out to interrogate specific interaction between NOX4 and key downstream signalling events, incorporating endoglin and SERPINE1 as identified NOX4-sensitive pro-angiogenic candidate targets whose expression was upregulated in NOX4OE versus EV diabetic CB-ECFCs (Fig. 5C, D). IPA of our RNA sequencing dataset generated from CB-ECFCs isolated from healthy donors and those with gestational diabetes (Fig. 4) indicated that NOX4 downregulation in diabetic CB-ECFCs may directly inhibit SERPINE1 expression with consequent reduction in angiogenic capacity (Fig. 6A). This analysis also highlighted likely reinforcement of SERPINE1 downregulation in diabetic CB-ECFCs through parallel activation of TP53 (p53) mediated by altered E2F signalling (identified by GSEA as significantly enriched in diabetic CB-ECFCs and linked with key functions; Fig. 4D–F) and endoglin expression, noting that the predicted direction of change of these two proteins was inconsistent with the dataset. As IPA indicated activation of p53 as a direct consequence of NOX4 downregulation in diabetic versus healthy CB-ECFCs (Fig. 6A), we investigated specific impact of NOX4OE on protein phosphorylation in diabetic CB-ECFCs, as an important regulator of activity and cell signalling. Consistent with our gene expression and network analyses, the presented original proteome profiler blots and heat map clearly show largely decreased phosphorylation of several proteins linked to key cellular processes in NOX4OE versus EV control (Fig. 6B, C). Notably, phosphorylation of p53 at serine 46 was amongst the most downregulated in NOX4OE diabetic CB-ECFCs (highlighted on original membranes in Fig. 6B, outlined in red in Fig. 6C), in keeping with its established inhibition of angiogenic signalling and pro-angiogenic effects of NOX4OE (Fig. 5B). Indeed, the observed decrease in p53 phosphorylation in response to NOX4OE was the largest with direct relevance to activation of angiogenic signalling, further demonstrating its importance in this context. Consistent with known negative impact of activated p53 on endoglin and E2F (and contrary to the direction of change predicted by IPA), decreased serine 46 phosphorylation at p53 (Fig. 6B, C) correlated with increased protein expression of all three candidate NOX4 targets in OE versus EV diabetic CB-ECFCs (Fig. 6D–G). Taken together, these data indicate that restoration of NOX4 levels in diabetic CB-ECFCs results in direct activation of a potent pro-angiogenic signalling response, mediated via specific downstream protein targets and regulatory networks, and rescued angiogenic capacity.

## Discussion

**Fig. 7:**
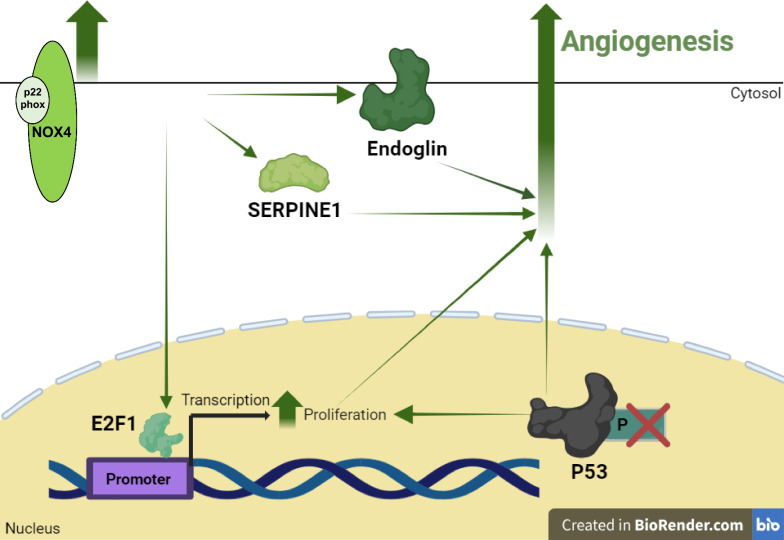
**Restoration of NOX4 expression in diabetic CB-ECFCs rescues angiogenic capacity by specifically inducing pro-angiogenic signalling.** Summary schematic indicating that restoration of NOX4 levels in diabetic CB-ECFCs leads to upregulated E2F1 signalling in parallel with fully restored angiogenic function. E2F1 is a transcription factor which positively regulates key genes associated with efficient progression through the cell cycle, DNA replication and subsequent proliferation, which is critical to support an efficient and potent pro-angiogenic response. NOX4 induction in diabetic CB-ECFCs also led to reduced phosphorylation of anti-proliferative P53 (at S46), increased expression of SERPINE1 and endoglin, which are implicated in pro-angiogenic signalling

Ischaemic CVD remains a leading cause of global morbidity and mortality which is strongly associated with reduced revascularisation capacity [39]. It is particularly prevalent in diabetic patients in whom EC dysfunction and tissue hypoxia represent critical determinants of disease progression [21]. It is well established that exposure of mature and progenitor ECs to uncontrolled hyperglycaemia drives angiogenic dysfunction and impaired homeostatic signalling, conferring elevated risk of ischaemia [15, 40]. ECFCs circulate in the blood and support physiological vascular function and are recruited to sites of vessel injury to promote active repair and regeneration [41]. Consequently, targeting of ECFC-dependent neovascularisation events in ischaemic tissue has emerged as an attractive therapeutic option, towards recovery of nutrient delivery and waste removal [5]. However, ECFCs are typically less abundant and dysfunctional in CVD patients and are further suppressed in diabetic tissue due to local hypoxia and hyperglycaemia, thereby limiting direct clinical translation. As such, there is clear need for development of innovative approaches to harness the therapeutic potential of circulating ECFCs to promote restoration of angiogenic capacity and resilience within the host disease microenvironment [16, 20, 42, 43]. In this regard, the current study presents convincing novel data indicating that restoration of NOX4 protein expression and downstream signalling in diabetic CB-ECFCs rescues angiogenic potential, thereby helping to address such barriers to translation. As summarised in Fig. 7, we show that plasmid NOX4OE leads to upregulation of pro-angiogenic factors (SERPINE1, endoglin) [38, 44], in parallel with increased expression of pro-proliferative E2F1 driven by NOX4-dependent p53 dephosphorylation and recovery of angiogenesis [30, 32]. Consistent with our previous report that NOX4 siRNA knockdown attenuated angiogenic capacity in healthy CB-ECFCs [10], which identified NOX4 as a central determinant of basal signalling and function, exposure of these cells to short-term high glucose culture resulted in loss of PMA-induced migratory and tube-forming responses in parallel with lack of NOX4 upregulation, despite minimal impact under basal conditions (Fig. 1). Taken together, these intriguing data implicate NOX4-mediated signalling as a critical regulator of angiogenic capacity in both experimental and clinical diabetes and highlight this novel axis as a potential target towards improving vasoreparative function and resilience of circulating ECFCs within diabetic patients who are highly susceptible to both onset and progression of ischaemic CVD.

We initially demonstrated that plasmid OE of NOX4 rescued ability of healthy CB-ECFCs to mediate PMA-dependent angiogenic response under hyperglycaemic conditions (Fig. 2), highlighting its potential as a therapeutic target. Based on these novel findings, we hypothesised that reduced expression of this key ROS-generating protein may contribute to attenuated capacity of clinically relevant diabetic CB-ECFCs to support angiogenesis. Whilst others have reported similar decreases in ECFC function [15, 16, 23] and implicated NOX4 as a pivotal regulator of altered EC homeostasis in diabetes [45, 46], here we present novel data indicating that reduced angiogenic capacity and eNOS activation, which is regulated by NOX4 [47], is associated with markedly decreased NOX4 protein expression in CB-ECFCs exposed to hyperglycaemia. Furthermore, and consistent with the findings of our PMA-induced experiments (Figs. 1 and 2) and previous study [10], highlighting a critical regulatory role for NOX4 in maintaining angiogenic function of healthy CB-ECFCs, NOX4OE in chronically-diabetic CB-ECFCs restored impaired angiogenic capacity. It is interesting to note that NOX4OE did not impact CB-ECFC migration and tubulogenesis with short-term high glucose exposure alone (Fig. 2), suggesting that exogenous or endogenous stimulation may be required for cell priming to support NOX4-induced enhancement of angiogenic capacity, as observed in response to both in vitro PMA treatment and exposure to in vivo stress linked with gestational diabetes (Figs. 2 and 5). Whilst several studies have indicated that genetic or pharmacological manipulation of diabetic ECFCs can reverse dysfunction, here we demonstrate for the first time specific significance of NOX4 NADPH oxidase in regulating CB-ECFC angiogenic signalling under both experimental and clinical disease conditions [16, 23, 26, 48, 49]. It is interesting to note that whilst our study indicates that NOX4 may protect ECFC angiogenic signalling in diabetes, EC-specific NOX4 expression is reported to drive associated microvascular dysfunction [46], as further evidence of the established complexity and context-dependent nature of ROS signalling [50].

Although gene or drug targeting approaches have been extensively investigated towards supporting repair of dysfunctional ECFCs prior to reintroduction to patient donors, autologous cell therapy strategies have significant limitations [20, 51]. It is becoming increasing apparent that direct targeting of endogenous circulating ECFCs within the patient is likely to be more effective, from both a therapeutic and practical perspective, than conventional autologous strategies. Gaining detailed understanding of ECFC signalling in both naïve diseased cells and in response to selective modification provides clear opportunity to inform innovative therapeutic interventions based on gene targeting or drug repurposing to reinstate vasoreparative capacity [52–54]. In this regard, we conducted transcriptomic profiling to determine key genes and pathways which are negatively impacted and linked with reduced angiogenic capacity in diabetic CB-ECFCs to define key mechanisms underlying NOX-dependent signalling. Pathway analysis highlighted significantly reduced ‘wound healing’ in diabetic CB-ECFCs, consistent with their intrinsic angiogenic dysfunction, whilst GSEA identified significant enrichment of ‘E2F targets’ as an established family of transcription factors which are essential for cell cycle progression and DNA replication [30]. This finding aligns with both reports that in vivo and in vitro hyperglycaemia promote oxidative stress, DNA damage and reduced repair, resulting in cell cycle arrest, programmed cell death and senescence [55–57], and our data indicating that diabetic CB-ECFCs possess decreased proliferative potential as a key contributor to angiogenic response [1, 23, 58]. Whilst previous studies have highlighted attenuated proliferation in diabetic ECFCs [23], we show that NOX4 drives E2F1 expression as a likely critical process underlying ECFC progression through the cell cycle. Taken together, these data indicate a central role for NOX4-dependent downstream signalling in protecting ECFC proliferative capacity in diabetes which may serve to promote heightened cell resilience and survival within hostile tissue environments.

Further to identification of enhanced pro-proliferative E2F signalling in NOX4OE diabetic CB-ECFCs, proteome profiler analysis indicated markedly elevated levels of pro-angiogenic proteins, endoglin and SERPINE1. Complementary network analysis incorporating these two targets highlighted likely parallel direct and indirect NOX4 dependent regulation of SERPINE1 and endoglin, respectively, both of which promote angiogenesis [44, 59, 60]. Endoglin is a major glycoprotein which is critical for maintaining efficient angiogenesis and vascular integrity, as evidenced by impaired embryonic development and survival observed in endoglin-deficient mice [37, 61]. Although many studies have reported a critical role for endoglin in regulating EC angiogenesis [62–65], only a few have noted its specific importance in ECFCs. For example, siRNA knockdown of endoglin in ECFCs attenuated vessel formation in Matrigel in vivo [61], whilst endoglin is required for protection of ECFC tubulogenic capacity and barrier function against TNFα-induced inflammatory stress [66]. Given recognition of the critical importance of endoglin in maintaining vascular homeostasis and mediating pathological angiogenesis [67, 68], it is likely that NOX4-dependent induction of endoglin in diabetic CB-ECFCs plays a pivotal role in promoting angiogenic signalling and rescue of angiogenic capacity. SERPINE1 (or plasminogen activator inhibitor-1) mediates enzymatic cleavage of plasminogen to plasmin which acts together with matrix metalloproteinases to promote extracellular matrix degradation [69]. Several studies have positively associated SERPINE1 expression with angiogenesis in various disease contexts, with specific linkage to EC migratory response [38, 60, 70], consistent with its apparent ability to mediate NOX4-dependent rescue of tube formation capacity in diabetic CB-ECFCs. Specific upregulation of SERPINE1 with NOX4OE in this context highlights this key remodelling protein as a potential intermediate target towards restoring vasoreparative capacity of endogenous dysfunctional ECFCs in diabetic patients.

Complementary network interrogation using IPA highlighted p53 phosphorylation status as a key downstream mediator of NOX4-dependent endoglin and E2F expression, in addition to direct regulation of SERPINE1 by NOX4. Indeed, phosphokinase proteome analysis indicated markedly reduced phosphorylation of p53 at S46 in diabetic CB-ECFCs subjected to NOX4OE in parallel with increased protein expression of endoglin, E2F and SERPINE1 as pro-angiogenic downstream targets. These data are consistent with previous reports that p53 activation is associated with reduced angiogenesis whilst inactivation confers beneficial impact on both EC function and CVD progression [71–74]. For example, pharmacological inhibition or genetic deletion of p53 in human cardiac microvascular ECs promoted in vitro angiogenesis, whilst mice with EC-specific deletion of p53 showed enhanced vessel formation following both hindlimb ischaemia and induction of experimental diabetes [71, 75]. Further studies in HUVECs indicated that p53 OE suppresses expression of KLF2 which is critical for eNOS induction [76], whilst p53 phosphorylation at S1177 specifically mediates eNOS inhibition [75, 77]. Indeed, accumulating evidence supporting p53 as a key pathogenic driver of vascular dysfunction [71, 75, 78] has led to its proposal as a CVD therapeutic target [75, 79]. Considered together with the findings of the current study, selective targeting of p53 phosphorylation in ECFCs could represent an innovative approach towards improved treatment and management of ischaemic CVD [26]. In this regard and further to its involvement in several diseases, a range of small molecule inhibitors of p53 (e.g. pifithrin, PFT-α) have been developed which show positive impacts in both in vitro and in vivo models of vascular disease [78, 80]. For example, treatment of mouse aortic ECs with PFT-α or siRNA against p53 attenuated oxidative stress, inflammation, and EC dysfunction induced by hyperglycaemia [81], whilst PFT-α administration promoted reparative angiogenesis in a rat ischaemic stroke model [82]. Identification of p53 as a central mediator of CB-ECFC angiogenic response in hyperglycaemia is therefore timely in further advancing understanding of its specific role in associated CVD [78]. Together with our other data, these findings represent important mechanistic insight into the specific role of ECFC NOX4 in determining angiogenic function in both experimental and clinical diabetes, whilst providing exciting opportunity for targeted intervention towards improved management of ischaemic CVD in diabetic patients.

## Conclusion

In summary, the findings of this study clearly implicate NOX4 signalling as a critical regulator of CB-ECFC angiogenic (dys)function in diabetes. Despite some progress towards advancing clinical application of ECFCs, major barriers to autologous cell therapy remain, including decreased proliferative potential, suppression of intrinsic angiogenic function in patients and limited efficacy within hostile diseased tissue environments. Combined with requirement for immune suppression to support allogeneic administration and reduced number of autologous ECFCs in prospective patient recipients, attention has focused on deciphering mechanisms underlying pro-angiogenic pathway dysregulation in the disease setting towards development of more viable strategies. In this regard, application of pharmacological agents to augment endogenous ECFC efficacy has emerged as a potential alternate therapeutic approach. In the context of our data using CB-ECFCs, it is important to highlight that although gestational diabetes is a transient phenomenon, cells from this origin are an established model of vascular dysfunction with direct relevance to chronic diabetes [23]. With a view towards heightened clinical application, future studies should consider impact of varying diabetes duration on NOX4 angiogenic signalling using peripheral blood ECFCs from adult donors at different disease stages, including consideration of likely influence of diabetes medication, whilst noting that these cells are technically challenging and difficult to both isolate and culture [15]. Nonetheless, the results presented in this manuscript build on our previous report that healthy NOX4OE ECFCs show enhanced angiogenic function and signalling in vitro and promote in vivo neovascularisation [10] to support selective manipulation of NOX4 and/or its downstream signalling targets as an innovative approach towards improving endogenous vasoreparative capacity in diabetes and basis for future translational studies. Taken together, as well as significantly advancing mechanistic understanding, these data highlight realistic opportunity to harness NOX4 pro-angiogenic signalling to overcome current translational barriers towards positively impacting key biological functions and limiting progression of ischaemic CVD.

## Data Availability

The original data are available from the corresponding author upon request. RNA sequencing data are available from GEO, accession number GSE296585.

## Abbreviations

ECFCs: Endothelial colony forming cells
CVD: Cardiovascular disease
NOX4: NADPH oxidase 4
CB-ECFC: Cord blood-derived ECFC
OE: Overexpression
PMA: Phorbol 12-myristate-13-acetate
eNOS: Endothelial nitric oxide synthase
E2F1: E2F transcription factor 1
EC: Endothelial cells
EGM2: Endothelial growth media
FBS: Foetal bovine serum
EV: Empty vector
DMEM: Dulbecco's modified eagle's medium
MTT: 3–4,5-Dimethylthiazol-2-yl-2,5-diphenyl-2H-tetrazolium bromide
DMSO: Dimethyl sulfoxide
HBSS: Hank’s balanced salt solution
BCA: Bicinchoninic acid
PVDF: Polyvinylidene fluoride
SDS: Sodium dodecyl sulphate
NOX2: NADPH oxidase 2
VEGF: Vascular endothelial growth factor
GSEA: Gene set enrichment analysis
VEGFR2: Vascular endothelial growth factor receptor 2
IPA: Ingenuity pathway analysis
ROS: Reactive oxygen species
KLF2: Krüppel-like factor 2
PFT-α: Pifithrin
siRNA: Small-interfering RNA
CTL: Control
DG: D-Glucose
VCL: Vehicle
S46: Serine 46
